# Modulation of social interactions by immune stimulation in honey bee, *Apis mellifera*, workers

**DOI:** 10.1186/1741-7007-6-50

**Published:** 2008-11-17

**Authors:** F-J Richard, A Aubert, CM Grozinger

**Affiliations:** 1 Departments of Entomology and Genetics, WM Keck Center for Behavioral Biology, Gardner Hall, North Carolina State University, Raleigh, NC 27695, USA; 2 Laboratoire Ecologie, Evolution, Symbiose, Université de Poitiers, 86000 Poitiers, France; 3 DESCO, Faculté des Sciences, Parc de Grandmont, 37200 Tours, France

## Abstract

**Background:**

Immune response pathways have been relatively well-conserved across animal species, with similar systems in both mammals and invertebrates. Interestingly, honey bees have substantially reduced numbers of genes associated with immune function compared with solitary insect species. However, social species such as honey bees provide an excellent environment for pathogen or parasite transmission with controlled environmental conditions in the hive, high population densities, and frequent interactions. This suggests that honey bees may have developed complementary mechanisms, such as behavioral modifications, to deal with disease.

**Results:**

Here, we demonstrate that activation of the immune system in honey bees (using bacterial lipopolysaccharides as a non-replicative pathogen) alters the social responses of healthy nestmates toward the treated individuals. Furthermore, treated individuals expressed significant differences in overall cuticular hydrocarbon profiles compared with controls. Finally, coating healthy individuals with extracts containing cuticular hydrocarbons of immunostimulated individuals significantly increased the agonistic responses of nestmates.

**Conclusion:**

Since cuticular hydrocarbons play a critical role in nestmate recognition and other social interactions in a wide variety of insect species, modulation of such chemical profiles by the activation of the immune system could play a crucial role in the social regulation of pathogen dissemination within the colony.

## Background

Defenses against pathogens and diseases have been remarkably well conserved across species, from invertebrates to vertebrates [1,2]. In particular, the innate immune system, which responds to bacteria, fungi, viruses and other parasites, is very similar even at the molecular level in such diverse phyla as mammals, insects, and even plants [3,4]. However, while molecular responses to pathogens have been the main focus of research, it has been demonstrated that behavioral responses could also serve a critical role in supporting the immune response to infection [5-7]. Research in mammals suggests that immune-stimulated individuals withdraw from their healthy social mates [8], which would represent an adaptive strategy to protect non-infected conspecifics [7]. However, it is still unclear whether alterations in social interactions represent a general and conserved mechanism implicated in the control of disease transmission within social groups.

Social insects, such as honey bees, ants and termites, are ideal models for studying mechanisms regulating disease transmission in social groups. Social insects provide an ideal environment for transmission of pathogens, since nests are typically environmentally controlled and there are hundreds if not thousands of potential hosts with frequent interactions, allowing for relatively easy dissemination [9-12]. Furthermore, based on modeling [13-15] and experimental studies [16,17], the spread of disease within a colony is strongly dependent on social interactions between workers. Interestingly, though honey bees are the target of a large number of pathogens [18-21], they have substantially fewer genes associated with the innate immune response pathways compared with solitary insect species [22]. This suggests that honey bees may have developed alternative strategies to deal with pathogens.

Social insects have evolved multiple behavioral adaptations to avoid or combat parasites and pathogen infections. One suite of behaviors involves removing infected nestmates, or avoiding the source of infection. *Zootermes angusticollis *termites in experimentally controlled contact with spores (*Metarhizium anisopliae*) emit a vibratory signal that repels nestmates from the source of infection [23]. *Coptotermes *termites wall off nestmates infected by nematodes (Fujii 1975 cited in [24]). Honey bees reduce the impact of pathogenic bacteria, fungi and parasitic mites through hygienic behaviors, where workers identify and remove infected larvae from the healthy brood [25,26]. Honey bees also can produce a 'social fever' where workers increase temperature in the brood patch to kill larval pathogens [27]. A second suite of behavioral responses to infection involves altering the behavior of the infected individual to reduce the transmission of the disease. Parasitized bumblebee workers stay outside overnight rather than returning to the nest and actively seek out colder temperatures [28], which delays the development of the parasite. *Nosema*-infected honey bee workers become foragers, no longer engage in in-hive tasks and brood care, and have less contact with the queen [29]. However, although these behavioral modifications may reduce the chances and/or speed of parasitic dissemination within the colony, they could also reflect some parasite manipulation of the host favoring its horizontal transmission to other colonies (for example, food-borne transmission [30]). A third behavioral modification involves altering social interactions between healthy and diseased nestmates. Increased contact between healthy and infected individuals can results in 'social' vaccination of the healthy individual, increasing its immunity [31]. Termites and ants in groups have decreased mortality from fungal infection compared with isolated individuals (for example, [31-33]), suggesting that social interactions such as grooming could have some important function. Virus-infected honey bee workers are the target of increased grooming [34]. However, increase social interactions could cause increase transmission as well [35]. In *Formica polyctena *ants there is increased social contact and allogrooming from untreated ants toward their immune-challenged conspecifics, resulting in reduced locomotion of the immune-challenged individual in cage studies [9]. Decreased locomotion of an infected individual could decrease the spread of pathogens in a colony [9,31]. These studies suggest that nestmates can distinguish healthy from unhealthy nestmates, although in many cases, colony level studies need to be performed to determine if the behavioral modifications truly decrease disease spread. Furthermore mechanisms by which nestmates distinguish diseased and healthy workers are unknown.

Chemical communication plays a key role in regulating social interactions in social insects. Recent data confirm that cuticular hydrocarbons are involved in worker interactions and discrimination [36-38], and are generally implicated in the inter- and intra-specific recognition in many different species, including ants [39,40], termites [41], wasps [42], and bees [43]. Social insects use cuticular hydrocarbons to distinguish between nestmates and non-nestmates, and to reject non-nestmate individuals [44,45]. Hydrocarbon profiles are highly sensitive to genotype [46,47], environment [38,48], physiological state [49] and age in social insects (reviewed in [49]), suggesting that they could also be altered by immune processes. Indeed, there is some evidence for chemical communication of immunological status to nestmates. Parasitization by an ectoparasitic mite (*Varroa destructor*) significantly alters the cuticular hydrocarbon profile of emerging adult bees [50], but it is still unknown whether or not such changes in chemical profiles are correlated with altered nestmate responses to parasitized bees, and if these chemical changes are caused by the host's immune responses or factors produced by the mite.

Here we experimentally stimulated the immune system of honey bees, *Apis mellifera*, by injecting bees with a solution of bacterial coat proteins, that is, lipopolysaccharides (LPS), which has the advantage of stimulating an immune response in the host without spreading any infectious agent to the healthy nestmates. LPS is commonly used to stimulate immune responses in insects, and activates the JNK pathway of the innate immune system (see [51,52]). Here, we confirmed immune system activation after LPS administration by measuring expression levels of an immune response gene (*Defensin2*) in the fat bodies of honey bee workers. We monitored social interactions between healthy honey bee workers and immune-challenged conspecifics. We analyzed the cuticular chemical profiles of immune-challenged and healthy individuals to determine if chemical communication can be modified by infection. Finally, we treated healthy worker bees with the cuticular extracts of control or LPS-injected workers and examined their interactions with untreated nestmates, to determine if the changes in the chemical profiles could indeed be responsible for the altered social interactions.

## Results

### Effects of LPS treatment on expression of *Defensin2*

**Figure 1:**
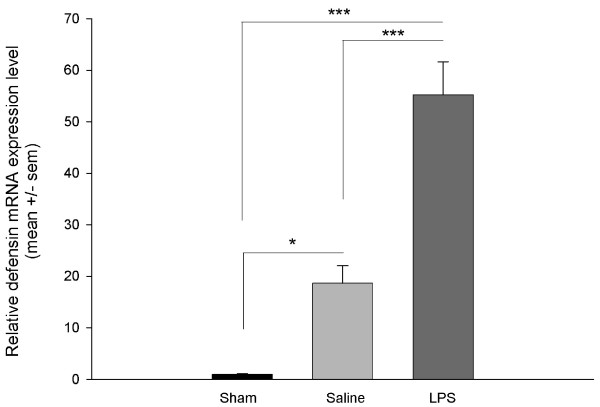
**Lipopolysaccharide injection activates the immune system of honey bees**. Relative expression levels of *Defensin 2 *(*Def2*), an immune response gene, are increased in the fat bodies of 7-day-old honey bee workers after saline- and lipopolysaccharide (LPS)-injection compared with untreated controls (sham). LPS-injection causes a significantly higher expression of *Def2 *than saline-injection. Relative expression levels of *Def2 *were monitored using quantitative real-time polymerase chain reaction using fat bodies from individual bees. Data were collected from two independent trials, and each trial used bees from a different source colony (sham: *N *= 18; saline: *N *= 16; LPS: *N *= 18). Figure shows the effects of treatments on mean ± standard error of the mean quantitative expression of *Def2 *RNA (F(2,46) = 31.702; *P *< 0.001). Post-hoc tests:* *P *< 0.05; *** *P *< 0.001.

We used quantitative real-time polymerase chain reaction (RT-PCR) to measure *Def2 *expression levels in the workers' fat bodies to determine if LPS injection activated expression of immune response genes. *Def2 *expression was significantly affected by treatment (F(2,46) = 33.2; *P *< 0.001; Figure 1). There were also significant differences between colonies (F(2,46) = 10.07; *P *= 0.002) but no significant colony*treatment interaction (F(2,46) = 2.56; *P *= 0.08). Although saline-injection causes a significant increase in *Def *levels compared with the sham bees, LPS-injected bees had significantly higher levels of *Def2 *than both sham and saline-injected bees (Tukey's honestly significant differences (HSD) post-hoc tests, *P *< 0.05). Thus, while rupturing the cuticle by injection still stimulates the immune system, the effect of LPS is significantly larger.

### Effects of LPS treatment on locomotion and mortality

Lethality of our treatments was assessed 8 hours after treatment. After treatment, two out of 12 of the sham individuals, three out of 11 of the saline-injected individuals, and three out of 11 of the LPS-injected bees died. There was no significant difference between the three treatment groups (Chi^2 ^= 0.485; df = 2; *P *> 0.78). No additional mortality was found 24 hours after treatment.

The behavioral effects of treatments (*N *= 8 for each treatment) were individually assessed on isolated bees placed in circular arenas for 5 minutes. Statistical analyses revealed no significant effects of treatments on general activity (that is, total time spent self-grooming: sham: 18.5 ± 17.5; saline-injected: 9 ± 17.6; LPS-injected: 9.5 ± 37.7 seconds) or locomotion (that is, number of lines crossed: sham: 127 ± 39; saline-injected: 137 ± 22; LPS-injected: 145 ± 37) (H(2)<0.98; *P *> 0.61).

### Effects of LPS treatment on nestmates' social interactions

**Figure 2:**
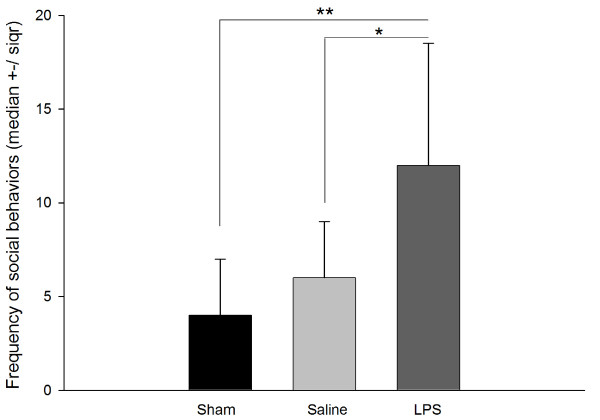
**Immunostimulated bees receive increased social interactions from untreated nestmates**. Bees were reared in cages, 10/cage until they were 7 days old. Individual bees were removed from cages and handled and treated with carbon dioxide (sham), injected with saline, or injected with lipopolysaccharides (LPS). After 4 hours, treated bees were returned to their original cage and non-agonistic social interactions (antennating, allogrooming) with untreated nestmates were monitored during a 5-minute period. Data were collected into two independent colonies (12 sham bees; 11 saline-injected bees; 12 LPS-injected bees). Figure shows effects of treatment on median ± the semi interquartile range frequency of social interactions (H(2) = 11.68; *P *< 0.002). Post-hoc tests: * *P *< 0.05, ** *P *< 0.01.

To monitor social interactions, we used a modified nestmate recognition assay in which a treated animal is placed back in a cage with its nestmates and social interactions are monitored. This assay has been used regularly in bees to examine nestmate recognition, and reliably reflects similar interactions in field colonies [44,53]. As above, there was no effect on locomotion of treated individuals during the social interaction assay (H(2) = 1.74; *P *= 0.41; data not shown). Concerning social interactions, agonistic and non-agonistic contacts were analyzed separately. Indeed, the calculation of a global 'aggression index' as it is commonly used in behavioral studies in insects would have been statistically irrelevant since agonistic behaviors (that is, opening mandibles, biting, gaster flexion, stinging) were virtually absent from our observations. Indeed, none of the 12 sham-treated, and only one out of 11 saline-injected and two out of 12 LPS-injected bees were attacked. Moreover, these attacks included only opening of mandibles and biting. Thus, although there was a slight trend for increased agonistic behavior toward LPS-injected bees, there was no overall significant difference in the proportion of agonistic interactions between the three groups (Chi^2 ^= 2.132; df = 2; *P *> 0.344). However, there were significant differences in the frequency of non-agonistic contacts (that is, antennating and allogrooming) of the healthy nestmates with individuals between our three experimental groups (H(2) = 11.68; *P *< 0.002, Figure 2). Post-hoc comparisons revealed that LPS-injected subjects received significantly more non-agonistic social contacts than sham or saline-injected workers (Dunn's post-hoc tests, *P *< 0.01 and *P *< 0.05 respectively; Figure 2).

Overall, these results show that LPS increased general social activities in healthy nestmates towards the immune-challenged insects, but did not induce a specific agonistic outcome.

### Effects of LPS treatment on cuticular hydrocarbon profiles

**Figure 3:**
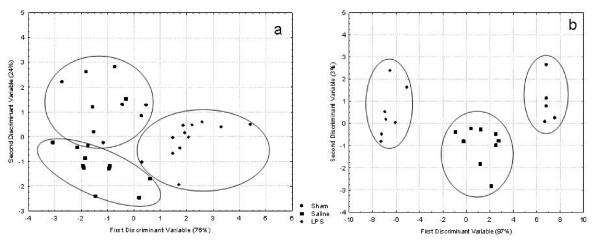
**Immunostimulated bees display significantly different cuticular hydrocarbon chemical profiles**. Discriminant analysis was performed on the cuticular chemical profiles of sham, saline- and lipopolysaccharide-injected honey bee workers based on their relative proportions of chemical compounds (a, colony SDI8, F(14,46) = 5.69, *P *< 0.0001). Data were obtained from gas chromatography analysis of cuticular washes. Similar results were obtained for bees derived from a different colony in a second independent trial (b, colony SDI11, (F(26,10) = 6.74; *P *= 0.0016).

Non-polar compounds were extracted from insects' whole bodies using pentane, and chemical profiles of cuticular extracts were analyzed using gas chromatography (GC). In our experimental conditions, all the chemical compounds we identified by mass spectrometry (MS) analysis were hydrocarbons: alkanes, alkenes, alkynes, and methyl-alkanes. The comparison of the cuticular profiles of sham, saline or LPS-injected workers 4 hours after injection revealed significant statistical differences in the overall chemical profiles of samples collected from colony SDI8 (F(14,46) = 5.69, *P *< 10^-4^, Figure 3a and Table 1). Mahalanobis chemical distances between both the sham/saline-injected and sham/LPS-injected bees were significantly different (MD = 5.02; *P *< 0.02 and MD = 12.07; *P *< 0.0001, respectively). Moreover, Mahalanobis chemical distance between LPS-injected bees and saline-injected bees was also significantly different (MD = 13.2; *P *< 0.0001). Table 1 summarizes the relative proportion of each chemical compound on the workers' cuticles; out of 32 compounds analyzed, significant changes in relative proportion were only found for seven compounds. Furthermore, no new compounds were found in the LPS-treated sample. Thus immunostimulation is associated with variation in the relative proportion of existing compounds.

**Table 1:** Effect of immuno-stimulation on cuticular hydrocarbon profiles

**Substance**	Sham	Saline	LPS	Kruskal-Wallis H(2, N = 33)
***Alkanes***				
Octadecane	0,17 ± 0,11	0,18 ± 0,05	0,19 ± 0,11	NS
Nonadecane	0,29 ± 0,14	0,35 ± 0,21	0,15 ± 0,04	NS
Eicosane	0,09 ± 0,04	0,09 ± 0,03	0,06 ± 0,04	NS
Heneicosane	1,24 ± 0,40	1,73 ± 0,65	0,84 ± 0,20	NS
Docosane	0,14 ± 0,02	0,16 ± 0,04	0,11 ± 0,02	NS
Tricosane	4,42 ± 0,73	4,70 ± 0,89	3,92 ± 0,39	NS
*Tetracosane*	tr	tr	tr	NS
Pentacosane	2,88 ± 0,66	2,76 ± 0,16	2,99 ± 0,17	NS
Hexacosane	0,38 ± 0,13	0,38 ± 0,07	0,34 ± 0,04	NS
Heptacosane	10,21 ± 3,91	9,76 ± 1,76	9,88 ± 1,45	NS
Octacosane	0,67 ± 0,06	0,62 ± 0,04	0,59 ± 0,06	NS
Nonacosane	14,15 ± 0,83	12,56 ± 1,52	12,33 ± 1,12	NS
Tritriacontane	0,72 ± 0,15	0,76 ± 0,06	0,77 ± 0,06	NS
Hentriacontane	17,24 ± 3,59	17,49 ± 1,99	18,70 ± 1,12	NS
Dotriacontane	0,23 ± 0,06	0,30 ± 0,08	0,30 ± 0,07	p = 0.05
Tritriacontane	3,03 ± 0,93	4,60 ± 1,51	4,51 ± 1,00	p = 0.024
***Alkenes***				
*Nonadecene*	tr	tr	tr	NS
Tricosene	0,30 ± 0,08	0,31 ± 0,06	0,29 ± 0,03	NS
Pentacosene	0,37 ± 0,06	0,35 ± 0,05	0,32 ± 0,03	p = 0.049
Heptacosene	0,39 ± 0,11	0,36 ± 0,13	0,26 ± 0,04	p = 0.001
Nonacosene	1,17 ± 0,09	0,96 ± 0,10	0,98 ± 0,08	p = 0.014
Hentriacontene Isomere1	4,42 ± 0,47	4,23 ± 0,21	4,37 ± 0,52	NS
Hentriacontene Isomere 2	6,17 ± 0,52	6,06 ± 0,24	6,28 ± 0,61	NS
Dotriacontene	0,89 ± 0,12	0,98 ± 0,04	1,02 ± 0,08	p = 0.026
Tritriacontene	16,77 ± 3,02	19,24 ± 0,49	20,30 ± 1,71	p = 0.01
***Alkynes***				
*Pentacosyne*	tr	tr	tr	NS
Tritriacontyne	2,08 ± 0,46	2,43 ± 0,83	2,44 ± 0,31	NS
***Methylalkanes***				
11,13,15-Methylpentacosane	0,30 ± 0,07	0,29 ± 0,07	0,38 ± 0,04	NS
11,13-Methylheptacosane	2,39 ± 0,31	2,60 ± 0,58	2,50 ± 0,21	NS
11,13,15-Methylnonacosane	2,15 ± 0,19	2,34 ± 0,56	2,27 ± 0,24	NS
11,13,15-Methylhentriacontane	1,31 ± 0,12	1,43 ± 0,35	1,42 ± 0,15	NS
11,13,15,17-Methyltritriacontane	0,56 ± 0,07	0,68 ± 0,17	0,69 ± 0,08	NS

Data for this figure were obtained from gas chromatography analysis of cuticular washes of worker bees from colony SDI8 (10 Sham bees, 11 Saline-injected bees, 12 LPS-injected bees). These data represent the relative proportions of each compound found across all three treatment groups. For the discriminant analysis, the 3 compounds in *italics *were not used for analyses, due to their very low proportion (<0.1%).

Similar results for the comparison of the cuticular profiles of sham, saline and LPS-injected bees were obtained with bees from a second colony (colony SDI11, F(26,10) = 6.74; *P *= 0.0016, Figure 3b and Additional file 1. Mahalanobis chemical distances between both the sham/saline-injected and sham/LPS-injected bees were significantly different (MD = 35.6; *P *< 0.001 and MD = 182.4; *P *< 0.0001, respectively). Moreover, Mahalanobis chemical distance between LPS-injected bees and saline-injected bees was also significantly different (MD = 63.8; *P *< 0.0001). However, different chemicals were found to show significant variations in relative proportion compared with colony SDI8 (Table 1 and Additional file 1). Thus, immunostimulation causes colony-specific variation in hydrocarbon cuticular proportions.

Since alkenes seem to play an important role in nestmate recognition in bees [37], we assessed the effect of treatments on the relative proportions of alkenes. Treatment had a significant effect on the relative proportion of the different alkenes between workers from colony SDI8 (F(12,50) = 3.6; *P *= 0.0007). Mahalanobis chemical distances between sham/saline-injected, sham/LPS-injected, and saline-injected/LPS-injected bees were significantly different (MD = 4.06; *P *< 0.02, MD = 6.51; *P *< 0.001, and MD = 4.03; *P *= 0.017, respectively). Similar results were obtained with colony SDI11 (F(10,26) = 2.37; *P *= 0.037). However, Mahalanobis chemical distances between sham/saline-injected, sham/LPS-injected bees were not significantly different (MD = 1.59; *P *> 0.05 and MD = 0.98; *P *> 0.05, respectively) but saline-injected/LPS-injected were significantly different (MD = 2.25; *P *= 0.03).

### Effect of immunostimulated nestmate cuticular extract on nestmate interactions

**Figure 4:**
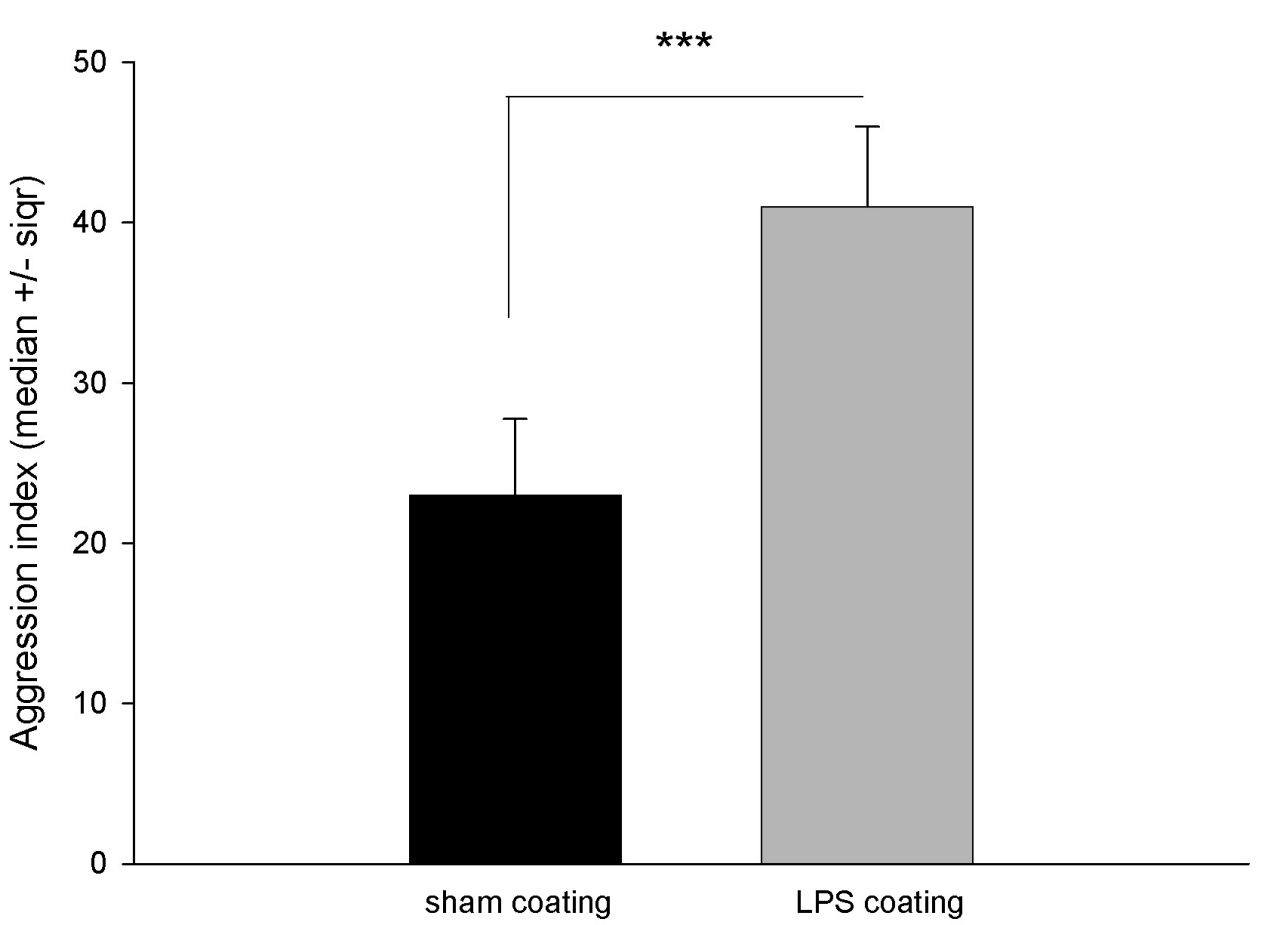
**Extracts of immunostimulated workers increases nestmate social interactions**. Bees were reared in cages, 15/cage until they were 7 days old. One naïve bee was removed from each cage and coated with 5 μl of extracts from sham or lipopolysaccharide (LPS)-treated bees (one bee equivalent). After the bees were coated, each one was returned to her original cage and social interactions with untreated nestmates were monitored during a 5-minute period. Data shown represent median ± the semi interquartile range values of the aggression index, calculated from measured social behaviors [38]. Treatment groups consisted of 12 bees coated with cuticular extracts of sham bees, and 12 bees coated with cuticular extracts from LPS-treated workers. 'LPS coating' induced significantly more interactions (agonistic) compared with the control (*U *= 10; *P *= 0.0001).

The following study was performed to determine if the changes in chemical profiles associated with immunostimulation are indeed sufficient to alter nestmate social interactions. Bees from a single colony were reared in cages as before. Sham or LPS-injected individuals were kept in isolation for 4 hours (as before) and then collected. Cuticular extracts were collected from these individuals and pooled according to treatment. A naïve bee from a new cage was then coated with 5 μl of extract (1 worker equivalent) from the cuticles of sham or LPS-injected workers. Coated bees were then placed back in their original cages, and social interactions were monitored as before. An 'aggression index' [38] was then calculated from the frequency of social behaviors observed. Indeed, the changes induced by coating generated a suitable amount of agonistic behaviors to allow a valid calculation of this behavioral index. Analyses revealed that coatings significantly altered social interactions in worker bees. The presence of a bee coated with cuticular extracts from LPS-injected workers induced significantly more agonistic behaviors (that is, a significantly higher aggression index) in nestmates, compared with a bee coated with cuticular extracts from sham bees (Mann and Whitney U = 10; *P *= 0.0001, Figure 4).

## Discussion

Using bacterial endotoxins (that is, LPS), we activated the innate immune system of individual insects, and monitored the responses of healthy nestmates. Although both LPS- and saline-injection caused an increase in expression of an immune response gene (as seen in previous studies [54]), this increase was substantially and significantly higher for the LPS-injected individuals, and only LPS-injection induced significant changes in social interactions between treated animals and their healthy conspecifics. The evaluation of the lethality and behavioral effects of LPS on treated honey bees confirm that the social changes observed were not the result of behavioral alterations in treated individuals. Furthermore, these differences in social interactions correlated with changes in cuticular hydrocarbon profiles induced by injection; again, these changes were greater for the LPS- versus saline-treated bees. Finally, we found that the coating of naïve bees with cuticular extracts from LPS-injected bees induced significant changes in social behaviors of healthy nestmates compared with controls. In sum, these results suggest that immune system activation can have significant effects on honey bee cuticular chemical profiles, which in turn results in changes in social interactions with nestmates.

Both injection with LPS and coating with extracts of an LPS-injected individual stimulated changes in social responses of healthy nestmates towards the treated individual. However, treatment with LPS elicited only increases in non-agonistic interactions (antennating, grooming) while coating with extract from LPS-injected individuals stimulated agonistic interactions (biting, stinging). Indeed, the coating of a social insect with exogenous chemicals is known to induce aggression in nestmates since this induces global changes in the chemical profile of the coated individual, although coating ants with the cuticular hydrocarbons of their nestmates does not elicit an aggressive response [55]. These results suggest that if the proportions of chemicals are changed, social interactions are modified. It is possible in our experiments that coating individuals with extracts caused additional changes to the chemical profiles that elicited a stronger behavioral response from the healthy nestmates. Regardless, both experiments confirm that LPS (injected or the associated changes in chemical profiles) alters social responses of healthy nestmates compared with controls.

The altered social interactions could benefit the individual, the nestmates or the pathogen. Grooming appears to play some crucial role in individual resistance to pathogens in social insects. For both termites and ants, individuals infected with a pathogenic fungus that were placed in groups are less likely to die than those reared in isolation [15,32,33]. Grooming was not specifically observed in either of these studies, although Hughes et al [33] found a decreased amount of fungus on the cuticle over time. However, this physical removal was observed in both isolated and group-reared termites. Virus-infected bees were found to be subjected to increased grooming [34]. Thus, ants, termites, and honey bees may all increase grooming of nestmates in response to a variety of pathogens. Furthermore, while grooming is clearly beneficial to the infected individual, it may also benefit the group by resulting in a 'social vaccination' of the naïve nestmate. Indeed, rearing naïve termites with termites previously exposed to pathogens can improve the subsequent resistance of the naïve termites to a challenge with a fungal pathogen [31]. This anticipatory immuno-activation could be induced by grooming, either through contact with small amounts of pathogen fragments, or with immuno-active molecules produced by the infected host and thus stimulating immune defenses of naïve conspecifics (reviewed in [9]). However, social interactions can obviously also increase the spread of pathogen. Further studies using full colonies and active pathogens will be necessary to determine the effects of behavioral modifications towards unhealthy nestmates on pathogen transmission.

Our studies suggest that immunostimulation causes changes in cuticular hydrocarbon patterns that result in altered social interactions. Upon immune stimulation, fat bodies and hematocytes secrete antimicrobial peptides and enzymes into the hemolymph [56]. As noted by Schal et al [57], an active association exists between hemolymph and the cuticle since compounds from the hemolymph are readily transported to the outer epicuticular surface. Indeed, hydrocarbons are produced by oenocytes and are transported through a hemolymph pathway to both external and internal tissues, including the epicuticle, fat bodies, and ovaries [58]. Our studies also suggest that the effects of immunostimulation do not cause addition of novel cuticular hydrocarbon compounds, and variation in these compounds appear colony specific. Thus, immunostimulation may alter existing biosynthesis or transport pathways, and thereby shift cuticular chemical profiles. Previous studies demonstrated that alkenes are important for nestmate recognition [37], and in our studies there were significant differences in the relative proportion of alkenes between saline-injected and LPS-injected bees from two different colonies. However, different specific alkenes were modulated by treatment in the two colonies, suggesting that immune stimulation does not produce a specific 'disease' signal pattern, but rather alters overall cuticular hydrocarbon patterns in a non-specific way that may differ for bees derived from different genotypic or environmental backgrounds. Alterations of cuticular hydrocarbons and pheromones by pathogens remain largely unexplored phenomena, but it could be relatively general. Indeed, parasitization by an ectoparasite mite (*Varroa destructor*) during larvae development significantly affects the relative proportion of cuticular hydrocarbons of emerging adult bees [50], while in mealworm beetles, male immunocompetence and parasite load appears to be communicated to females via changes in sex pheromones [59,60].

## Conclusion

Here, we provide clear evidence of altered behaviors in healthy individuals toward immuno-stimulated conspecifics in honey bees. Previous studies on mammalian species such as mice or rats demonstrated a social disinterest or withdrawal of immune-stimulated individuals [8], and this has been argued to represent an adaptive strategy to protect healthy conspecifics [7]. In our studies, immune stimulation leads to the increased tendency of healthy individuals to enter into contact with their infected nestmate. This could lead to an increase in transmission of the pathogen, decreased transmission by slowing the movement of infected individuals, physically reduce the pathogen or parasite load on the infected individual, or allow 'social vaccination' of the healthy nestmates. Further studies will be necessary to determine the effect of the observed behavioral modification on pathogen transmission. Our study also suggests that immunostimulated individuals signal their status to healthy nestmates via changes in cuticular hydrocarbon profiles. Since chemical communication regulates social interactions in a wide variety of species, including insects and vertebrates, modulation of chemical cues by the immune system may play an important and general role in the regulation of social behaviors.

## Methods

### General bee rearing

Honey bee colonies were maintained according to standard commercial procedures at North Carolina State University's Honey Bee Research Facility. Bees were derived from colonies headed by queens instrumentally inseminated with semen from a single, different drone (*Apis mellifera carnica*, from Glenn Apiaries, CA, US [61]); thus, due to the haplodiploidy of honey bees, the coefficient of relatedness among the offspring in each colony was 0.75. Since cuticular hydrocarbon profiles and nestmate interactions are very sensitive to genotypic variation [46,47], using highly related bees within individual trials was important for our studies. Four colonies were used for these studies: SDI8, SDI11, SDI72, and SDI73. The specific source colonies used for the different experiments are noted in each experimental section.

To provide bees of known age, frames containing late-stage pupae were removed and placed in a 33°C incubator for adult bees to emerge. One-day-old workers were placed in 10-cm diameter Petri dishes (15 bees/dish), and dishes were kept in a humidity (40% relative humidity) and temperature (33°C)-controlled environmental chamber under red light. Bees were fed a 50% sucrose/water solution, which was changed every 2 days. Bees were also treated with 0.1 queen bee equivalents of queen mandibular pheromone (QMP) (Pherotech, Canada). Every day, 10 μl of QMP were placed on a microscope slide and allowed to evaporate before being placed in the cage. This amount of QMP mimics a live queen in assays of worker behavior and physiology [62-64], and thus should help simulate normal rearing conditions. When the honeybee workers were 7 days old, individual bees were subjected to the following treatments: sham (handled and anesthetized with carbon dioxide, but not injected), saline injected (anesthetized with carbon dioxide and injected with a physiological solution), LPS injected (anesthetized with carbon dioxide and injected with a solution of LPS, see below for details). Injected bees were maintained individually in separate Plexiglas cages (10 × 10 × 7 cm^3^) and fed 50% sucrose ad libitum for 4 hours after injection, before being collected or used in behavioral assays. For the behavioral assays, the injected bees were subjected to a modified nestmate recognition assay [65]; these assays were performed with colony SDI11, colony SDI72, and colony SDI73. For the cuticular hydrocarbon analysis, injected bees were collected on to dry ice 4 hours after treatment; these assays were performed with bees from colony SDI11 and colony SDI8 (in two separate trials). For the gene expression analysis, injected bees were collected on to dry ice 4 hours after treatment; these assays were performed with bees from colony SDI11 and colony SDI8 in two separate trials. See below for detailed methods.

### Bacterial LPS solution

LPS is the major outer surface membrane component present in almost all Gram-negative bacteria and acts as an extremely strong stimulator of innate or natural immunity in diverse eukaryotic species ranging from insects to humans [66,67]. Based on previous trials and pilot studies, LPS-treatment consisted of a 4 μl injection of 500 ng/μl LPS (from *Escherichia coli *serotype 055:B5, Sigma) diluted in physiological (saline) solution (130 mM NaCl, 6 mM KCl, 4 mM MgCl_2_, 5 mM CaCl_2_, 160 mM sucrose, 25 mM glucose, 10 mM 4-(2-hydroxyethyl)-1-piperazineethanesulfonic acid in distilled water, pH 6.7, 500 mOsmol). Injections were performed into the abdominal cavity through tergites with a nano-injector equipped with a glass needle (Schley Compact Model II Instrument; Honey Bee Insemination Services, Davis, CA, US), using a binocular microscope for guidance (Leica MZ6 stereomicroscope).

### Expression level of *Defensin2 *(*Def2*) in abdominal fat body

We next tested if LPS treatment activated the expression of immune response genes in the fat bodies of 7-day-old worker bees, 4 hours after injection. Previous studies demonstrated that RNA expression of the antimicrobial peptide Defensin was significantly increased in honey bee workers upon injection with *E. coli *(reference [54]; and references therein). In our studies, we focused on *Defensin2 *since this gene was more highly up-regulated in microbe-injected honey bee larvae than *Defensin1 *[68]. Abdomens were dissected under RNAlater (Qiagen, Valencia, CA, US) to remove intestines, and the remaining cuticles with the associated fat bodies were collected. Total RNA was isolated from the fat bodies using an RNeasy RNA extraction kit (Qiagen, Valencia, CA, US), yielding 0.6–1.5 μg/individual bee. cDNA was synthesized from 150 ng RNA using Arrayscript reverse transcriptase (Ambion, Foster City, CA, US). Expression levels of *Defensin2 *were measured using quantitative RT-PCR (qRT-PCR) with an ABI Prism 7900 sequence detector and the SYBR green detection method (Applied Biosystems, Foster City, CA, US). *eIF3-S8*, a housekeeping gene that did not vary in expression levels in previous bee microarray studies [62,69] was used as a loading control. For each sample, triplicate qRT-PCR reactions were performed and averaged. A standard curve was generated for each primer using dilutions of genomic DNA to calculate the relative quantities of RNA levels for each sample. A dissociation curve and negative control (cDNA reaction without RT-enzyme) were used to ensure primer specificity and lack of contamination. Primers were designed using PrimerExpress software (Applied Biosystems, Foster City, CA, US). The sequences for the primers (5' to 3') used are as follows:

*Def2*-F: CAGAATTGATGGATTCCAACGA3;

*Def2*-R: CGCACGTTACCCTTCGATGT;

*eIFS8*-F: TGAGTGTCTGCTATGGATTGCAA;

*eIFS8*-R: TCGCGGCTCGTGGTAAA;

We evaluated the fat body gene expression levels of seven sham bees, seven saline-injected, and seven LPS-injected bees from colony SDI11, and 11 sham bees, nine saline-injected and 11 LPS-injected bees from colony SDI8. For each individual fat body sample, the quantities of *Def2 *and *eIF3-S8 *were calculated based on the standard curve. The ratio of the relative quantity of *Def2 *RNA levels (based on the standard curve) to that of the control gene (*eIF3-S8*) was calculated for each sample.

### Behavioral assessment of the deleterious effects of treatment

To determine whether LPS induced any deleterious effects in treated insects, we conducted a preliminary study in 7-day-old honey bees (*N *= 8 per treatment, from colony SDI8). Lethality and behavioral alterations (that is, changes in grooming and mobility) were individually monitored 8 hours after treatment. To assess mobility, a cross was drawn under the circular arena and the number of lines crossed during 5 minutes was used as a locomotion index. We also recorded lethality after the nestmate interaction assays (see below).

### Behavioral effects of treatments in nestmate interactions

To assess the effect of immune stimulation on nestmate interactions, an individual worker bee was picked up from the Petri dish where she was raised (10 bees/dish were used for these experiments), marked with a dot of enamel paint on the thorax and subjected to one of the three treatments: sham, saline-injected or LPS-injected. After the injection, each individual bee was isolated in a Plexiglas cage (10 × 10 × 7 cm^3^) for 4 hours. Then, the worker was reintroduced to the original Petri dish. Studies were conducted blind to the treatment group. Each cage was used only once.

Beginning 30 seconds after the reintroduction, the number and type of social behaviors of the non-treated bees toward the treated bee were recorded through a scanning method, every 30 seconds for 5 minutes. Two types of behavior were observed: non-agonistic social contacts (that is, antennal contacts, allogrooming) and agonistic interactions (that is, mandibular openings, bites, gaster flexions, stinging). Moreover, as it is commonly used in behavioral studies, an index of locomotor activity was used to assess the effects of treatments on the subjects' mobility. This index was obtained as described above. Mortality of the injected individual was recorded as well.

Behavioral assays involved bees from three different colonies. Groups consisted of 12 sham (five from colony SDI11, four from colony SDI72, and three from colony SDI73), 11 saline-injected (five from colony SDI11, four from colony SDI72, and three from colony SDI73), and 12 LPS-injected (five from colony SDI11, four from colony SDI72, and two from colony SDI73) subjects.

### Cuticular hydrocarbons analysis

We next tested if LPS-treatment altered the cuticular hydrocarbon profiles. Seven-day-old bees were collected 4 hours after the injection, as described for the behavioral assays. Each worker bee was submerged in 1 ml in pentane for 10 minutes to extract non-polar cuticular compounds. All the samples were maintained in the freezer at -20°C. After solvent evaporation, the samples were diluted in 50 μl of pentane with C16 as an internal standard (0.4 μg/μl).

GC was used to measure the relative proportions of the cuticular hydrocarbons in all of the samples. The analysis was performed on a HP 5890 equipped with capillary column (30 m × 0.25 mm inner diameter, 0.5 μm film thickness) DB-5 column (J&W Scientific, Folsom, CA, US) in splitless mode. Helium was used as the carrier gas at a head pressure of 18 psi (flow rate, 1.3 ml/minute). The oven temperature was held at 150°C for 2 minutes, increased to 250°C at 15°C/minute, increased to 300°C at 5°C/minute, and held for 20 minute. Injector and flame ionization detector temperatures were set at 300°C and 300°C, respectively.

For identification of the compounds present in the hydrocarbon profiles, one sample was subjected to GC/MS. A 2 μl portion was analyzed by splitless capillary GC/MS, using a Hewlett-Packard 6890 and a model 5973A msd with an electron impact ion source, HP-5ms (5% diphenyl-95% dimethylsiloxane) capillary column, 30 m × 0.25 mm inner diameter, 0.25 μm film thickness. Here, the oven temperature was held at 80°C for 2 minutes, increased to 200°C at 15°C/minute, increased to 300°C at 5°C/minute, and held for 15 minutes.

Under our extraction method, only hydrocarbons were apparent in the extract. We evaluated the cuticular chemical profiles of 10 sham (non-injected bees), 11 saline-injected and 12 LPS-injected bees from colony SDI8 and in a second trial compared bees (*N *= 5 for sham, *N *= 9 for saline-injected, and *N *= 7 for LPS-injected bees) from colony SDI11.

### Effect of immuno-stimulated nestmate cuticular extract on social interactions between nestmates

To determine if the changes in cuticular chemical profile associated with immunostimulation was indeed associated with the observed behavioral assays, we tested the effects of coating naïve bees with cuticular extracts from LPS-injected bees (or sham bees for controls). Bees from a single colony were reared in Petri dishes as above (SDI33). Two Petri dishes were set up a day before the dishes used for the behavioral assay. Bees from these cages were treated with either carbon dioxide (sham) or injected with LPS, as above, and were collected 4 hours after treatment. The cuticular extraction was performed as previously described; all the extracts for each treatment were pooled.

For the behavioral assay, all bees were marked on their thorax with a dot of enamel paint, except for the bee used for the behavioral assay. The test bee was removed from the cage and coated with 5 μl of a solution (sham cuticular extract or LPS-injected cuticular extract), which represent one bee equivalent. After full evaporation of the solvent, the bee was reintroduced into her original cage and observed every 15 seconds for 5 minutes. Non-agonistic social contacts (that is, antennal contacts, allogrooming) and agonistic interactions (that is, mandible openings, bites, gaster flexions, stinging) were observed and compiled to calculate an 'aggression index' [38]. Indeed, as stated earlier, the social changes induced by coating generated a suitable amount of agonistic behaviors for a valid calculation of this behavioral index, which represents a valuable tool, commonly used in studies of insect behavior.

The entire experiment was repeated in a second trial, using bees from colony SDI33. Treatment groups consisted of 12 bees coated with sham extracts and 12 bees coated with extracts from LPS-treated bees.

### Statistics

For the gene expression studies, the *Def2*/*eIF3-S8 *ratios for each individual were normalized to the average value for the sham-treated group for each colony, and a parametric analysis of variance (ANOVA) was performed with colony, treatment and colony*treatment interaction as variables, followed by post-hoc pairwise comparisons using Tukey's HSD tests. For the behavioral assays, owing to sample size and behavioral heterogeneity, non-parametric procedures were used to assess significant variations in our data [70]. Lethality of treatments was analyzed with a Chi^2 ^test. Comparisons of social interactions between sham, saline-injected, and LPS-injected bees were performed using the non-parametric Kruskal-Wallis ANOVA on ranks for global comparison, followed by Dunn's analyses for multiple comparisons. For assessing cuticular hydrocarbon profile similarity, a stepwise discriminant analysis was employed using Statistica 6.0. (StatSoft^® ^Inc.) Prior to analysis, each peak area was standardized according to Reyment (1989) [71]. Only peaks that were reliably and reproducibly quantifiable were used; peaks that were consistently below 0.1% of the total quantity were omitted. The chemical distance between each group was estimated as the Mahalanobis distance obtained from the discriminant analysis. GC analysis of cuticular hydrocarbons was conducted as in [38]. The effect of the treatment (sham, saline-injected, and LPS-injected) on the compound proportions was evaluated with a non-parametric Kruskal-Wallis ANOVA on ranks for global comparison.

## Abbreviations

ANOVA: analysis of variance; GC: gas chromatography; HSD: honestly significant differences; LPS: lipopolysaccharides; MS: mass spectrometry; QMP: queen mandibular pheromone; RT-PCR: real-time polymerase chain reaction.

## Authors' contributions

Behavioral tests, cuticular hydrocarbons analysis and gene expression analysis were performed by FJR. Statistical analyses were performed by FJR and AA. FJR, AA, and CMG were involved in the interpretation of the data and preparation of the manuscript. All authors read and approved the manuscript.

## Supplementary Material

Additional file 1 **table s1 mod.doc**. Click here for file
