# Supplementary material for: Modulation of social interactions by immune stimulation in honey bee, Apis mellifera, workers
Source: BMC Biol. 2008 Nov 17;6:50. doi: 10.1186/1741-7007-6-50 (PMC2596086; doi:10.1186/1741-7007-6-50)
Supplement: Additional file 1 — table s1 mod.doc. [file 1741-7007-6-50-S1.doc]

| **Substance** | Sham | | | Saline | | | LPS | | | Kruskal-Wallis H(2, N=21) |
| --- | --- | --- | --- | --- | --- | --- | --- | --- | --- | --- |
| ***Alkanes*** |  |  |  |  |  |  |  |  |  |  |
| Pentadecane | 0,08 | ± | 0,01 | 0,09 | ± | 0,02 | 0,08 | ± | 0,03 | NS |
| Heptadecane | 0,04 | ± | 0,01 | 0,04 | ± | 0,01 | 0,03 | ± | 0,00 | NS |
| Octadecane | 0,01 | ± | 0,00 | 0,01 | ± | 0,00 | 0,01 | ± | 0,01 | NS |
| Nonadecane | 0,12 | ± | 0,04 | 0,21 | ± | 0,03 | 0,11 | ± | 0,03 | NS |
| Eicosane | 0,02 | ± | 0,00 | 0,02 | ± | 0,01 | 0,02 | ± | 0,01 | NS |
| Heneicosane | 0,69 | ± | 0,06 | 0,86 | ± | 0,05 | 0,73 | ± | 0,04 | NS |
| Docosane | 0,11 | ± | 0,02 | 0,11 | ± | 0,02 | 0,11 | ± | 0,02 | NS |
| Tricosane | 3,56 | ± | 0,83 | 3,41 | ± | 0,79 | 3,63 | ± | 0,47 | NS |
| Tetracosane | 0,32 | ± | 0,04 | 0,12 | ± | 0,02 | 0,20 | ± | 0,09 | NS |
| Pentacosane | 14,94 | ± | 3,91 | 4,87 | ± | 0,78 | 8,27 | ± | 3,99 | NS |
| Hexacosane | 1,15 | ± | 0,17 | 0,64 | ± | 0,02 | 0,74 | ± | 0,25 | NS |
| Heptacosane | 34,44 | ± | 5,36 | 22,97 | ± | 1,96 | 28,16 | ± | 7,85 | NS |
| Octacosane | 0,61 | ± | 0,05 | 0,82 | ± | 0,04 | 0,88 | ± | 0,08 | p=0.02 |
| Nonacosane | 10,58 | ± | 2,19 | 15,99 | ± | 3,07 | 11,89 | ± | 1,11 | NS |
| Tritriacontane |  | - |  |  | - |  |  | - |  |  |
| Hentriacontane |  | - |  |  | - |  |  | - |  |  |
| Dotriacontane |  | - |  |  | - |  |  | - |  |  |
| Tritriacontane | 0,35 | ± | 0,23 | 0,74 | ± | 0,13 | 1,04 | ± | 0,39 | NS |
| ***Alkenes*** |  |  |  |  |  |  |  |  |  |  |
| Nonadecene | 0,08 | ± | 0,05 | 0,10 | ± | 0,02 | 0,07 | ± | 0,01 | NS |
| Tricosene | 1,94 | ± | 0,72 | 1,67 | ± | 0,59 | 0,80 | ± | 0,47 | NS |
| Pentacosene | 0,91 | ± | 0,57 | 0,50 | ± | 0,28 | 1,47 | ± | 0,67 | NS |
| Heptacosene | 2,63 | ± | 0,96 | 0,68 | ± | 0,59 | 1,02 | ± | 0,58 | NS |
| Nonacosene | 2,46 | ± | 0,84 | 2,49 | ± | 0,35 | 2,11 | ± | 0,39 | NS |
| Hentriacontene Isomere1 | 2,66 | ± | 0,48 | 4,55 | ± | 0,96 | 3,22 | ± | 1,19 | NS |
| Hentriacontene Isomere 2 | 2,57 | ± | 0,32 | 5,02 | ± | 1,29 | 3,59 | ± | 1,28 | NS |
| Hentriacontene Isomere 3 | 4,70 | ± | 0,57 | 9,00 | ± | 0,84 | 8,55 | ± | 2,09 | NS |
| Dotriacontene |  | - |  |  | - |  |  | - |  |  |
| Tritriacontene | 4,94 | ± | 0,88 | 9,94 | ± | 1,24 | 6,44 | ± | 3,11 | p=0.05 |
| ***Alkynes*** |  |  |  |  |  |  |  |  |  |  |
| Pentacosyne | 0,08 | ± | 0,01 | 0,11 | ± | 0,04 | 0,13 | ± | 0,06 | NS |
| Tritriacontyne | 0,82 | ± | 0,02 | 1,85 | ± | 0,43 | 1,14 | ± | 0,40 | NS |
| ***Methylalkanes*** |  |  |  |  |  |  |  |  |  |  |
| 11,13,15-Methylpentacosane | 0,49 | ± | 0,14 | 0,60 | ± | 0,15 | 0,57 | ± | 0,15 | NS |
| 11,13-Methylheptacosane | 2,72 | ± | 0,46 | 4,54 | ± | 1,01 | 3,46 | ± | 1,21 | NS |
| 11,13,15-Methylnonacosane | 3,24 | ± | 0,75 | 4,70 | ± | 0,89 | 3,76 | ± | 0,93 | NS |
| 11,13,15-Methylhentriacontane | 1,97 | ± | 0,50 | 2,29 | ± | 0,90 | 2,27 | ± | 2,59 | NS |
| 11,13,15,17-Methyltritriacontane | 0,69 | ± | 0,16 | 0,81 | ± | 0,13 | 0,75 | ± | 0,21 | NS |

**Table S1. Effect of immunostimulation on cuticular hydrocarbon profiles.** Data for this figure were obtained from a gas chromatography analysis of cuticular washes from workers from colony SDI11 (Sham=5 bees, Saline-injected=9 bees, LPS-injected=7 bees). These data represent the relative proportions of each compound found across all three treatment groups.
